# *TERT* Gene Fusions Characterize a Subset of Metastatic Leydig Cell Tumors

**DOI:** 10.1016/j.clgc.2021.02.002

**Published:** 2021-02-18

**Authors:** Bozo Kruslin, Zoran Gatalica, Ondrej Hes, Faruk Skenderi, Markku Miettinen, Elma Contreras, Joanne Xiu, Michelle Ellis, Elena Florento, Semir Vranic, Jeffrey Swensen

**Affiliations:** 1 Clinical Department of Pathology and Cytology “Ljudevit Jurak”, University Hospital Centre “Sestre milosrdnice”, Zagreb, Croatia; 2 School of Medicine, University of Zagreb, Zagreb, Croatia; 3 Caris Life Sciences, Phoenix, Arizona; 4 Department of Pathology, University of Oklahoma College of Medicine, Oklahoma City, Oklahoma; 5 Department of Pathology, Charles University, Medical Faculty and Charles University Hospital Plzen, Pilsen, Czech Republic; 6 Department of Pathology, Clinical Center, University of Sarajevo, Sarajevo, Bosnia and Herzegovina; 7 Laboratory of Pathology, Center for Cancer Research, National Cancer Institute, Bethesda, Maryland; 8 College of Medicine, QU Health, Qatar University, Doha, Qatar; 9 Biomedical and Pharmaceutical Research Unit, QU Health, Qatar University, Doha, Qatar

**Keywords:** Sex cord–stromal tumors, Molecular profiling, Sequencing, Targeted therapy, Leydig cell tumors

## Abstract

Metastatic Leydig cell tumors (LCT) are rare, difficult to treat malignancies without known underlying molecular-genetic events. We profiled 27 LCT cases using NGS and immunohistochemistry. Our study identified *TERT* gene fusions as a main genetic alteration and a potential therapeutic target in LCT. TOP1 and AR expressions may guide decisions on chemo- and/or hormone therapy for selected individual patients.

**Objective:**

Metastatic Leydig cell tumors (LCT) are rare, difficult-to-treat malignancies without known underlying molecular–genetic events. An index case of metastatic LCT showed an *LDLR–TERT* gene fusion upon routine genetic profiling for detection of therapeutic targets, which was then followed by an investigation into a cohort of additional LCTs.

**Patients and Methods:**

Twenty-nine LCT (27 male and 2 female patients) were profiled using next-generation sequencing and immunohistochemistry.

**Results:**

*TERT* gene fusions were detected only in testicular metastatic LCTs, in 3 of 7 successfully analyzed cases (*RMST:TERT,LDLR:TERT*, and *B4GALT5:TERT*). *TOP1* and *CCND3* amplifications were identified in the case with a *B4GALT5:TERT* fusion. A *TP53* mutation was detected in 1 metastatic tumor without a *TERT* fusion. Five primary (4 testicular and 1 ovarian) LCTs showed multiple gene amplifications, without a consistent pattern. A single metastatic ovarian LCT showed *BAP1* mutation and copy number amplifications affecting the *NPM1, PCM1*, and *SS18* genes. At the protein level, 4 of 7 metastatic and 6 of 10 primary testicular LCTs overexpressed Topo1. Androgen receptor was overexpressed in 10 of 13 primary testicular tumors and 2 of 5 metastatic testicular LCTs (without detectable ARv7 messenger RNA or ARv7 protein). Only 1 metastatic testicular LCT exhibited a high tumor mutational burden; all tested cases were microsatellite instability stable and did not express programmed cell death ligand 1.

**Conclusions:**

Our study for the first time identified *TERT* gene fusions as a main genetic alteration and a potential therapeutic target in metastatic LCTs. Topo1 and androgen receptor may guide decisions on chemotherapy and/or hormone therapy for selected individual patients.

## Introduction

Sex cord–stromal tumors are an uncommon group of neoplasms affecting gonads. In the testis, these tumors represent 4% of all neoplasms and are the second largest group of primary tumors after germ cell tumors.^1^ In ovary, sex cord–stromal tumors constitute 5% of all neoplasms, and 7% of malignant ovarian neoplasms belong to this group.^2^ Leydig cell tumors (LCT) are the most common pure form of sex cord–stromal tumor followed by Sertoli cell, granulosa cell, and pure stromal tumors.^1^ Little is known about the pathogenesis of these neoplasms beyond their rare association with germline fumarate hydratase mutations (hereditary leiomyomatosis and renal cell carcinoma syndrome, OMIM#150800) or the activating mutations that affect luteinizing hormone receptor in the pediatric population.^1^ In addition, DICER1 mutations have been reported in sporadic and hereditary ovarian sex cord stromal tumors.^3–6^ *DICER1* gene mutations have been implicated in the dysregulation of the steroid hormone synthesis including androgen receptor (AR).^7^

Molecular profiling studies on these tumors are sparse owing to the overwhelmingly benign course of the disease and curative surgical resection.^8^ A recent whole exome sequencing study of Yuan et al^8^ revealed that LCTs frequently harbor somatic mutations of *CDC27* (53%), *DICER1* (21%), and *MUC22* (21%) genes. Metastatic LCTs are clinically challenging and without a consensus treatment approach.

We have previously characterized multiple cancers with a comprehensive molecular profiling approach that uses various molecular techniques for the identification of potentially targetable biomarkers.^9–13^ Our initial case of metastatic LCT showed an *LDLR:TERT* gene fusion upon routine genetic profiling for the detection of therapeutic targets. This finding led us to investigate a cohort of additional LCTs.

## Materials and Methods

### Samples for the Study

Twenty-nine LCTs from 5 participating institutions (University Hospital Centre “Sestre milosrdnice,” Zagreb, Croatia; Caris Life Sciences, Phoenix, AZ; University of Oklahoma College of Medicine, Oklahoma City, OK; Charles University Hospital Plzen, Pilsen, Czech Republic; and National Cancer Institute, Bethesda, MD) were included in the current study.

Before molecular testing, each LCT case underwent confirmation of the histologic diagnosis and a review of the diagnostic immunohistochemical workup performed at the referring/participating pathology laboratories. For the study, all histopathologic reports and remnant LCT tissue samples provided by the referring laboratories and participating institutions were de-identified. Based on this, the study was compliant with 45 CFR 46.101(b) and was deemed exempt from institutional review board (IRB) approval and consent requirements were waived.

All molecular assays were performed at a CLIA/CAP/ISO15189/NYSDOH certified clinical laboratory (Caris Life Sciences, Phoenix, AZ).

### Immunohistochemistry

PD-L1 expression was assessed in the tumor cells and immune cells using SP142 antibody (Ventana Medical Systems, Oro Valley, AZ). PD-L1 expression was considered positive if either tumor cell or immune cells exhibited any membranous or cytoplasmic staining.^11,14^ AR (clone 441, Leica Biosystems, Buffalo Grove, IL) was analyzed using a 10% or higher threshold for nuclear positivity.^12–14^ The ARv7 splice variant was explored at the protein level by immunohistochemistry (EPR15656; Abcam) and at the messenger RNA level using anchored multiplex polymerase chain reaction for targeted RNA sequencing (ArcherDX).^9,11^ Topoisomerase 1 (Topo1) expression (clone 1D6; Leica Biosystems, Nussloch, Germany) was scored as 0+, 1+, 2+, or 3+ depending on the staining intensity, and the percent tumor stained was also recorded. The threshold for Topo1 overexpression was a staining intensity of 2+ or higher in 30% or more of the cancer cells.^15^

### Next-Generation Sequencing

The LCT samples were profiled using next-generation sequencing (NGS) of exons from 592 genes (SureSelect XT, Agilent, Santa Clara, CA; and the NextSeq instrument, Illumina, San Diego, CA). A full gene panel is available in the Supplemental Table 1.

For the study, the tumor mutational burden (TMB) was assessed by calculating the number of non-synonymous missense mutations, excluding common germline variants, per one megabase of DNA. TMB was considered high if 10 or more mutations/megabase (muts/Mb) were detected.^16^

Microsatellite instability (MSI) was calculated from the NGS data by direct analysis of short tandem repeat tracts in the target regions of sequenced genes. The count only included alterations that resulted in increases or decreases in the number of repeats; high MSI was defined as 46 or more altered microsatellite loci. This threshold was established by comparing NGS with the polymerase chain reaction–based microsatellite fragments analysis results from approximately 2100 samples.^10^

Copy number amplifications were assessed by comparing the depth of detected NGS sequence reads to calibrated control values. Genes having 6 or more copies were considered amplified.

The ArcherDx FusionPlex Assay (ArcherDX, Boulder, CO) was used for gene fusion assessment. The gene fusions panel (*n* = 54) is available in the Supplemental Table 2.

## Results

### Clinicopathologic Characteristics of the Cohort

Twenty-seven testicular (7 metastatic and 20 primary tumors) and 2 ovarian LCT (1 metastatic and 1 primary) were investigated. The mean patient age was 55.5 years (range, 23–94 years) for male patients; the 2 female patients with ovarian LCT were 45 and 69 years of age. The metastatic sites of testicular LCT included lung, liver, mediastinum, parasternal region, and retroperitoneum (×3). The only ovarian metastatic LCT site was peritoneum (4 years after the original ovarian tumor diagnosis).

### Immunohistochemical Biomarkers

**Fig. 1:**
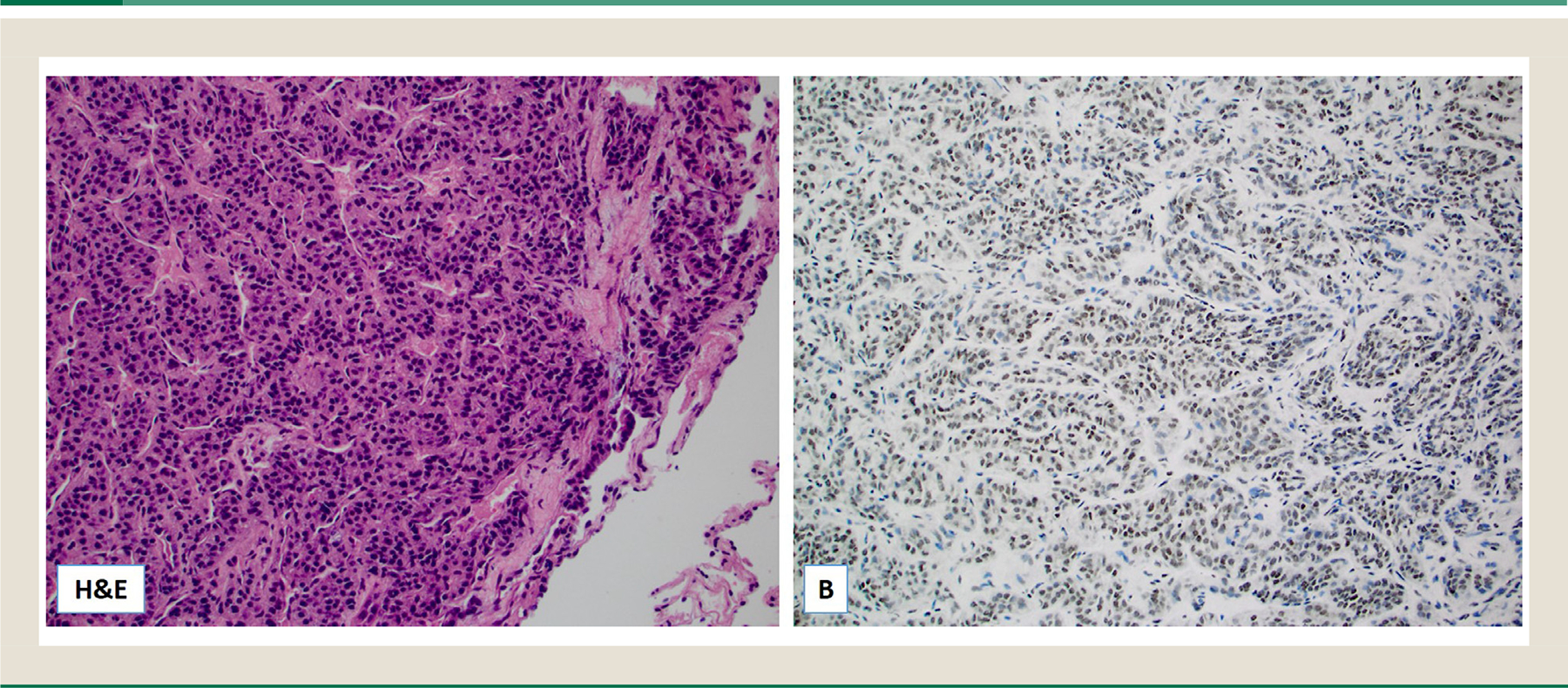
Hematoxylin and eosin (H&E) slide of a metastatic Leydig cell tumor to the lung (A); the tumor cells were diffusely positive for Topo1 by immunohistochemistry (original magnification ×20).

TOP1 was assessed in 17 testicular LCT: 6 of 10 primary (60%) and 4 of 7 metastatic (57%) LCT were positive (Table 1 and Fig. 1). Intriguingly, a single *TOP1* amplified testicular LCT showed no Topo1 protein expression by immunohistochemistry. AR expression was more prevalent among the primary testicular LCT (10/13) compared with the metastatic cases (2/5). All cases were ARv7 negative (at either the messenger RNA or protein levels).

### Genomic Characteristics of LCT

**Fig. 2:**
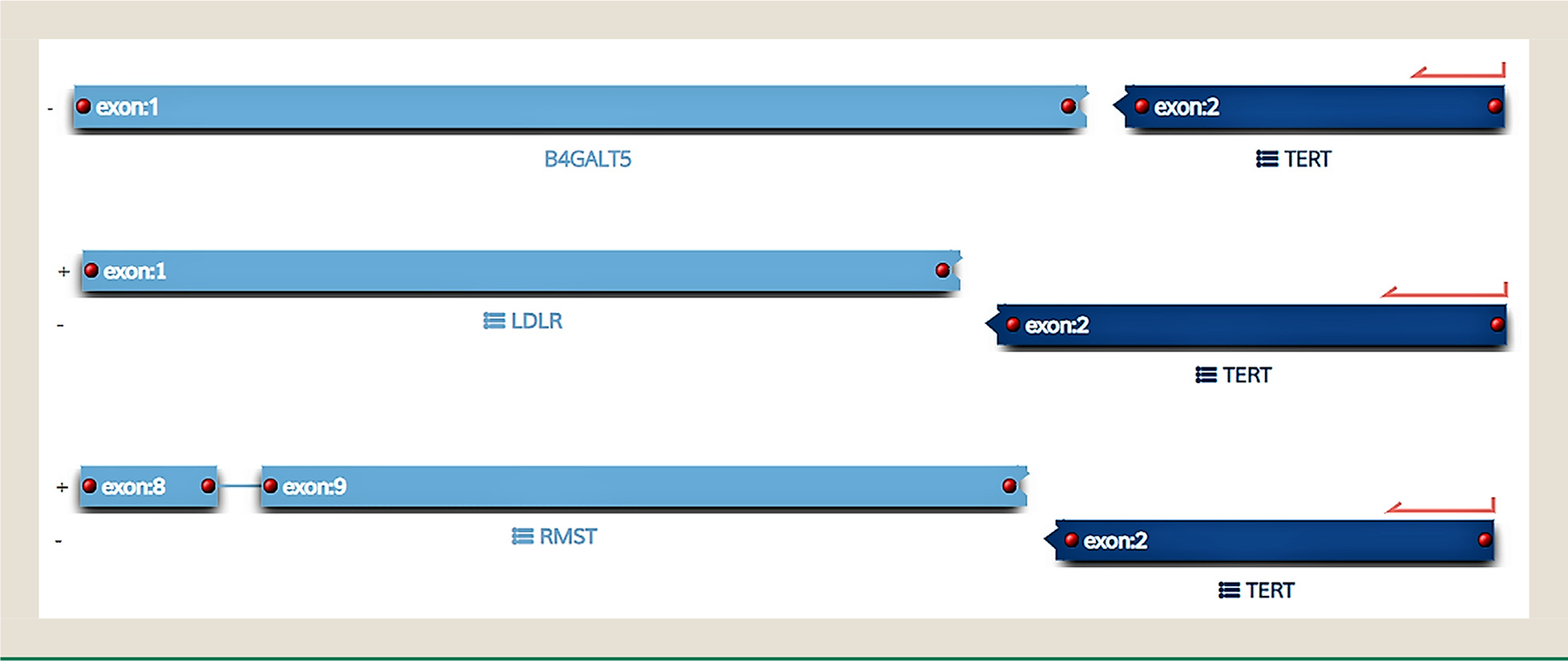
Telomerase reverse transcriptase (*TERT*) gene fusions detected in 3 metastatic (malignant) Leydig cell tumors of the testis.

Telomerase reverse transcriptase (*TERT*) gene fusions were exclusively seen in 3 of 7 successfully analyzed metastatic testicular LCT. The following fusions were detected: *RMST:TERT, LDLR:TERT*, and *B4GALT5:TERT* (Fig. 2). The specimen harboring the *B4GALT5:TERT* fusion also showed amplifications (>6 copies) of the *TOP1* and *CCND3* genes (Table 1). Neither of the 2 ovarian LCT harbored *TERT*-related fusions. *TERT* promoter mutations were not tested, because this region was not covered in the available commercial NGS panel at the time.

A NGS mutational profile was available for 15 testicular cases, which showed inconsistent and rare pathogenic mutations: 2 primary LCT harbored *CTNNB1* gene mutations (encoding *β*-catenin protein); *FOXO4* mutations were also observed in 2 cases (1 primary and 1 metastatic case), and a *TP53* mutation was observed in 1 metastatic LCT. All other mutations were detected in single cases (*NBN, MTOR, BAP1, MEN1,* and *CREBBP*) (Table 1). A single metastatic ovarian LCT had a *BAP1* mutation and copy number amplifications of the *NPM1, PCM1*, and *SS18* genes.

Copy number amplifications were detected in 8 of 18 successfully tested cases (6 testicular and 2 ovarian LCTs). The more prevalent copy number amplifications included those affecting *CCND3* (2 testicular) and genes in the fibroblast growth factor family: *FGF3* (1 primary ovarian), *FGFR3* (1 primary testicular and 1 primary ovarian), and *FGFR4* (1 metastatic testicular) (Table 1).

### Immuno-oncology Biomarkers

PD-L1 expression (threshold ≥1%) in the tumor cells or immune cells was not seen in any of the 15 tested testicular LCTs. All cases were MSI stable. A low tumor mutation burden (4–7 muts/Mb) characterized most of the testicular LCT except the peculiar metastatic case with a *B4GALT5:TERT* fusion and *TOP1* and *CCND3* amplifications that exhibited 11 muts/Mb (Table 1).

## Discussion

Our study represents the first comprehensive molecular study to examine potentially targetable molecular alterations in LCT including its malignant variants. One of the key findings in our study was that *TERT* gene fusions were a major detected genetic alteration in malignant, metastatic LCTs. This finding is novel and had not been previously reported in sex cord–stromal tumors, including LCT.^3,5,8^ In addition, all 3 described gene fusions affecting *TERT* gene have not been previously reported in the literature (review of the literature covered PubMed/MEDLINE and COSMIC database). TERT activity plays a central role in the unlimited self-renewal potential of cancer cells via telomerase activity that maintains telomere ends through addition of telomere repeats TTAGGG).^17^ This mechanism is considered one of the hallmarks of cancer.^18^ Various genomic alterations including *TERT* promoter mutations, rearrangements, amplifications, fusions, and promoter methylation have been well-characterized across human cancers.^19–21^ Limited information on the therapeutic implications of *TERT* genomic alterations are currently available. One recent in vitro study conducted on acral melanoma cells revealed the cytotoxic effects of TERT inhibitors in melanoma cells harboring *TERT* genomic alterations.^21^

The family of topoisomerase enzymes (TOP1 and TOPO2) are the key players in unwinding coiled DNA to facilitate the cell replication and transcription.^22^ Given their active role in DNA replication and transcription, several classes of drugs targeting TOP1 and TOPO2 have been developed. One of these drugs is camptothecin against TOP1, whose derivatives irinotecan and topotecan have been widely used as cytotoxic drugs in a clinical setting. Topo1 protein overexpression has been described in various cancers,^15^ whereas *TOP1* gene amplification is a much rarer event in cancers (the highest amplification rate [>10%] was reported in gallbladder, esophageal, and gastroesophageal carcinomas).^15^ Our study revealed a common (50%–60%) Topo1 protein expression in both primary and metastatic LCT, whereas *TOP1* gene amplification was observed in 1 metastatic case. This finding may be clinically relevant for malignant LCTs and provide a rationale for the treatment with camptothecin derivatives alone or combined with novel anticancer treatments such as antibody–drug conjugates that contain irinotecan.

Hormone therapy with antiandrogens has been used therapeutically in prostate cancer patients.^23^ Some of the commonly used antiandrogens (eg, bicalutamide) competitively inhibit ligand binding to the active AR. Our study also confirmed AR activity in LCTs without the presence of splice variant ARv7. In prostate cancer cells, ARv7 stems from aberrant messenger RNA splicing of AR exons 1 to 3, loss of exons 4 to 8, and inclusion of cryptic exon 3 into the transcribed *AR* gene.^24,25^ Consequently, the affected protein is constitutively active in the absence of androgens and facilitates the growth of prostate cancer in the presence of antiandrogens.^26,27^ We found AR expression in 40% of metastatic LCT without the ARv7 splice variant, which indicates a potential for treatment with antiandrogens.

Immunotherapy with immune checkpoint inhibitors against PD-1/PD-L1 has markedly improved the treatment and outcome of multiple solid and hematological cancers (eg, non–small cell lung carcinoma, melanoma, renal cell carcinoma, urothelial bladder carcinoma, triple-negative breast carcinoma, and classical Hodgkin lymphoma). Several currently available predictive biomarkers (PD-L1 expression, high TMB, and high MSI status) with approved clinical usefulness have been explored in this study. In contrast with testicular germ cell tumors,^28,29^ we found no PD-L1 expression in LCTs. With the exception of 1 case with high TMB (11 muts/Mb), all cases exhibited a low TMB, and all cases were MSI stable. Based on these results, it is unlikely that these patients would benefit from targeted therapy from immune checkpoint inhibitors.

There are several limitations of our study. There was a lack of matched primary sample analysis for cases with *TERT* fusion-positive metastases to determine whether the fusions represent early events in more aggressive cancers or later events associated with metastasis. If the fusions are early events, patients with fusion-positive primary tumors could have increased surveillance. In addition, the *TERT* promoter mutations, commonly observed in other malignancies (eg, gliomas, bladder, thyroid cancers, and melanoma), were not possible to examine in this study owing to the lack of the gene promoter coverage in the NGS panel available at the time of study.^30–33^ Finally, there is lack of feedback information on the usefulness of molecular profiling in the treatment of metastatic LCT with potentially actionable findings detected in our cohort (eg, overexpression of Topo1 and AR).

In conclusion, we identified for the first time *TERT* gene fusions as a main genetic alteration and several potential therapeutic targets in malignant, metastatic LCTs, including Topo1 and AR, which may help guide decisions on chemotherapy and/or hormone therapy for selected individual patients.

## Supplementary Material

33741265_MarkkuMiettinen_Supplefigs1

33741265_MarkkuMiettinen_Supplefigs2

## Figures and Tables

**Table 1:** Molecular Findings in the Leydig Cell Tumors Cohort

Biomarkers (number)	Testis (*n* = 27)	
	Primary ( *n* = 20)	Metastatic (*n* = 7)
Topo1*α* protein* (IHC) (*n* = 17)	6/10 (60%)	4/7 (57%)
AR* (*n* = 18)	10/13 (77%)	2/5 (40%)
ARv7 (NGS and IHC) (*n* = 18)	All ARv7 negative (mRNA or protein)	All ARv7 negative (mRNA or protein)
	**Genomic alterations**	
*TERT* gene fusions (NGS)^†^ (*n* = 19)	0/12 (0%)	3+/7 (43%): *LDLR:TERT* *B4GALT5:TERT* *RMSTTERT*
Mutational profile (NGS) (*n* = 15)	*CTNNB1* (2/10), *NBN* (1/10), *MTOR* (1/10), *FOXO4* (1/10), *BAP1* (1/10), *MEN1* (1/10), *CREBBP* (1/10)	*TP53* (1/5) *FOXO4* (1/5)
Copy number amplifications (NGS) (*n* = 16)	*MDM2, TCF3, LRIG3, HMGA2, CYP2D6, ASPSCR1* (1 case) *CDKN1B, DAXX, DDX5, PER1, VEGFB* (1 case) *MDM2, CDK4, CCND3, TFEB* (1 case) *GATA3, FGFR3, AKT2, TLX1, PIK3R2, MEF2B, JAK3, ERCC2, ELL, CIC, CD79A, CBFA2T3, BCL3* (1 case)	*TOP1, CCND3, MCL1* (1 case) *FGFR4, FLT4* (1 case)
	**I-O biomarkers**	
PD-L1 expression* (*n* = 15)	0/10 (0%)	0/5 (0%)
TMB (*n* = 7)	4–7/Mb (*n* = 4)	4–11/Mb (*n* = 3)
MSI (*n* = 7)	Stable (*n* = 4)	Stable (*n* = 3)
**Biomarkers**	**Ovary (*n* = 2)**	
	**Primary (*n* = 1)**	**Metastatic (*n* = 1)**
	
Topo1*α* protein*	Not available	Not available
AR*	Not available	Not available
	**Genomic alterations**	
*TERT* gene fusions (NGS)^†^	Absent	Absent
Mutational profile (NGS)	None	*BAP1*
Copy number amplifications (NGS)	*FGF3, FGFR3*	*NPM1, PCM1, SS18*
	**I-O biomarkers**	
PD-L1 expression*	Not available	Not available
TMB	Not available	3 mutations/Mb
MSI	Not available	Not available

* Assessed by IHC.

† Archer FusionPlex assay; *TERT* promoter region was not covered by the analysis.AR, androgen receptor; CAN, copy number amplifications; IHC, immunohistochemistry; I-O, immuno-oncology; mRNA, messenger RNA; MSI, microsatellite instability; NGS, next-generation sequencing; PD-L1, programmed cell death ligand 1; TERT, telomerase reverse transcriptase; TMB, tumor mutational burden.

## Data Availability

The data presented in the current study are available from the corresponding authors upon reasonable request.

## Clinical Practice Points

- Metastatic Leydig cell tumors are rare and difficult to treat malignancies without known underlying molecular-genetic events.
- We identified *TERT* gene fusions exclusively in malignant, metastatic Leydig cell tumors.
- Additional predictive biomarkers (Topo1 and AR) may help guide decisions on chemo- and/or hormone therapy for selected individual patients.
