# Supplementary material for: TERT Gene Fusions Characterize a Subset of Metastatic Leydig Cell Tumors
Source: Clin Genitourin Cancer. Author manuscript; Available in PMC 2023 Feb 8. (PMC9907364; doi:10.1016/j.clgc.2021.02.002)
Supplement: 33741265_MarkkuMiettinen_Supplefigs1 [file NIHMS1863327-supplement-33741265_MarkkuMiettinen_Supplefigs1.pdf]

Next-generation Sequencing (NGS) (Caris Life Sciences)

Point Mutations, Indels and Copy Number Alterations* (DNA)

ABL2	BRCA1	CREB3L1	ETV5	GID4 (C17orf39)	KNL1	NACA	PIM1	SDHC	TNFRSF17
ACSL3	BRCA2	CREB3L2	ETV6	GMPS	KRAS	NCKIPSD	PML	SDHD	TOP1
ACSL6	BRIP1	CREBBP	EWSR1	GNA13	KTN1	NCOA1	PMS2	SEPT9	TP53
ADGRA2	BUB1B	CRKL	EXT1	GNAQ	LCK	NCOA2	POLE	SET	TPM3
AFDN	CACNA1D	CRTC1	EXT2	GNAS	LCP1	NCOA4	POT1	SETBP1	TPM4
AFF1	CALR	CRTC3	EZH2	GOLGA5	LGR5	NF1	POU2AF1	SETD2	TPR
AFF3	CAMTA1	CSF1R	EZR	GOPC	LHFPL6	NF2	PPARG	SF3B1	TRAF7
AFF4	CANT1	CSF3R	FANCA	GPHN	LIFR	NFE2L2	PRCC	SH2B3	TRIM26
AKAP9	CARD11	CTCF	FANCC	GRIN2A	LPP	NFIB	PRDM1	SH3GL1	TRIM27
AKT2	CARS	CTLA4	FANCD2	GSK3B	LRIG3	NFKB2	PRDM16	SLC34A2	TRIM33
AKT3	CASP8	CTNNA1	FANCE	H3F3A	LRP1B	NFKBIA	PRKAR1A	SMAD2	TRIP11
ALDH2	CBFA2T3	CTNNB1	FANCG	H3F3B	LYL1	NIN	PRRX1	SMAD4	TRRAP
ALK	CBFB	CYLD	FANCL	HERPUD1	MAF	NOTCH2	PSIP1	SMARCB1	TSC1
APC	CBL	CYP2D6	FAS	HGF	MALT1	NPM1	PTCH1	SMARCE1	TSC2
ARFRP1	CBLB	DAXX	FBXO11	HIP1	MAML2	NR4A3	PTEN	SMO	TSHR
ARHGAP26	CCDC6	DDR2	FBXW7	HMGA1	MAP2K1 (MEK1)	NSD1	PTPN11	SNX29	TTL
ARHGEF12	CCNB1IP1	DDX10	FCRL4	HMGA2	MAP2K2 (MEK2)	NSD2	PTPRC	SOX10	U2AF1
ARID1A	CCND1	DDX5	FGF10	HNRNPA2B1	MAP2K4	NSD3	RABEP1	SPECC1	USP6
ARID2	CCND2	DDX6	FGF14	HOOK3	MAP3K1	NT5C2	RAC1	SPEN	VEGFA
ARNT	CCND3	DEK	FGF19	HSP90AA1	MCL1	NTRK1	RAD50	SRGAP3	VEGFB
ASPSCR1	CCNE1	DICER1	FGF23	HSP90AB1	MDM2	NTRK2	RAD51	SRSF2	VTI1A
ASXL1	CD274 (PDL1)	DOT1L	FGF3	IDH1	MDM4	NTRK3	RAD51B	SRSF3	WDCP

ATF1	CD74	EBF1	FGF4	IDH2	MDS2	NUP214	RAF1	SS18	WIF1
ATIC	CD79A	ECT2L	FGF6	IGF1R	MEF2B	NUP93	RALGDS	SS18L1	WISP3
ATM	CDC73	EGFR	FGFR1	IKZF1	MEN1	NUP98	RANBP17	STAT3	WRN
ATP1A1	CDH11	ELK4	FGFR1OP	IL2	MET	NUTM1	RAP1GDS1	STAT4	WT1
ATR	CDK4	ELL	FGFR2	IL21R	MITF	PALB2	RARA	STAT5B	WWTR1
AURKA	CDK6	EML4	FGFR3	IL6ST	MLF1	PAX3	RB1	STIL	XPA
AURKB	CDK8	EMSY	FGFR4	IL7R	MLH1	PAX5	RBM15	STK11	XPC
AXIN1	CDKN1B	EP300	FH	IRF4	MLLT1	PAX7	REL	SUFU	XPO1
AXL	CDKN2A	EPHA3	FHIT	ITK	MLLT10	PBRM1	RET	SUZ12	YWHAE
BAP1	CDX2	EPHA5	FIP1L1	JAK1	MLLT3	PBX1	RICTOR	SYK	ZMYM2
BARD1	CHEK1	EPHB1	FLCN	JAK2	MLLT6	PCM1	RMI2	TAF15	ZNF217
BCL10	CHEK2	EPS15	FLI1	JAK3	MNX1	PCSK7	RNF43	TCF12	ZNF331
BCL11A	CHIC2	ERBB2 (HER2/NEU)	FLT1	JAZF1	MRE11	PDCD1 (PD1)	ROS1	TCF3	ZNF384
BCL2L11	CHN1	ERBB3 (HER3)	FLT3	KDM5A	MSH2	PDCD1LG2 (PDL2)	RPL22	TCF7L2	ZNF521
BCL3	CIC	ERBB4 (HER4)	FLT4	KDR (VEGFR2)	MSH6	PDGFB	RPL5	TET1	ZNF703
BCL6	CIITA	ERC1	FNBP1	KEAP1	MSI2	PDGFRA	RPN1	TET2	
BCL7A	CLP1	ERCC2	FOXA1	KIAA1549	MTOR	PDGFRB	RPTOR	TFEB	
BCL9	CLTC	ERCC3	FOXO1	KIF5B	MYB	PDK1	RUNX1	TFG	
BCR	CLTCL1	ERCC4	FOXP1	KIT	MYC	PER1	RUNX1T1	TFRC	
BIRC3	CNBP	ERCC5	FUBP1	KLHL6	MYCN	PICALM	SBDS	TGFBR2	
BLM	CNTRL	ERG	FUS	KMT2A (MLL)	MYD88	PIK3CA	SDC4	TLX1	
BMPR1A	COPB1	ESR1	GAS7	KMT2C (MLL3)	MYH11	PIK3R1	SDHAF2	TNFAIP3	
BRAF	CREB1	ETV1	GATA3	KMT2D (MLL2)	MYH9	PIK3R2	SDHB	TNFRSF14	
