# Supplementary material for: TERT Gene Fusions Characterize a Subset of Metastatic Leydig Cell Tumors
Source: Clin Genitourin Cancer. Author manuscript; Available in PMC 2023 Feb 8. (PMC9907364; doi:10.1016/j.clgc.2021.02.002)
Supplement: 33741265_MarkkuMiettinen_Supplefigs2 [file NIHMS1863327-supplement-33741265_MarkkuMiettinen_Supplefigs2.pdf]

Fusions panel (n=54) (Caris Life Sciences)

ABL	BRD3	ETV5	INSR	MYB	NUMBL	PRKCA	RSPO3
AKT3	BRD4	ETV6	MAML2	NOTCH1	NUTM1	PRKCB	TERT
ALK	EGFR	EWSR1	MAST1	NOTCH2	PDGFRA	RAF1	TFE3
ARHGAP26	ERG	FGFR1	MAST2	NRG1	PDGFRB	RELA	TFEB
AXL	ESR1	FGFR2	MET	NTRK1	PIK3CA	RET	THADA
BCR	ETV1	FGFR3	MSMB	NTRK2	PKN1	ROS1	TMPRSS2
BRAF	ETV4	FGR	MUSK	NTRK3	PPARG	RSPO2	
